# Synthesis, characterization and sorption studies of a zirconium(iv) impregnated highly functionalized mesoporous activated carbons†

**DOI:** 10.1039/c9ra10103a

**Published:** 2020-04-06

**Authors:** Sonalika Sonal, Prem Prakash, Brijesh Kumar Mishra, G. C. Nayak

**Affiliations:** Department of Environmental Science and Engineering, Indian Institute of Technology (Indian School of Mines) Dhanbad-826004 India brijesh@iitism.ac.in +91-9471191704; Department of Applied Chemistry, Indian Institute of Technology (Indian School of Mines) Dhanbad-826004 India

## Abstract

This study aimed to develop a highly functionalized adsorbent material for the removal of persistent anionic reactive dye. The modification process was commenced *via* a wet oxidation method by using zirconium salt as an impregnating material. The process led to an increase in the overall porosity, thermal stability and its oxidative functionality. The newly synthesised material was named ZrAC. The morphological and textural images revealed the irregular and eroded structures with an increase in porosity of the modified adsorbent. The results of chemical and spectral analysis disclosed that the material had successfully gained the oxidative functionality over the surface that will favour the removal of anionic dye. Equilibrium isotherms and adsorption kinetics studies insinuate that the overall process of adsorption follows the Sips isotherm and pseudo-second order kinetic model, respectively. The monolayer adsorption capacity of ZrAC was found to be superior (506 mg g^−1^) to AC at 500 mg L^−1^ concentration of persistent reactive dye. Moreover, the desorption capabilities of ZrAC were found to be more prominent, which finally affirms its potential use in a continuous flow system as a reusable adsorbent. Additionally, the stability of zirconium, corroborated from ICP-MS and XPS data, revealed the stability of zirconium after adsorption cycles thus verified its reusability. Thus, the characterization and experimental results of ZrAC strongly advocated its potential as a future adsorbent for removal of reactive dyes.

## Abstract 2

The present work focuses to develop a varied meso-microporous sorbent by using Zr(iv) as an impregnated metal. The modification develops the oxidative functionalities and porosity of the sorbent that enhances its efficiency for the removal of RB19.

## Graphical abstract

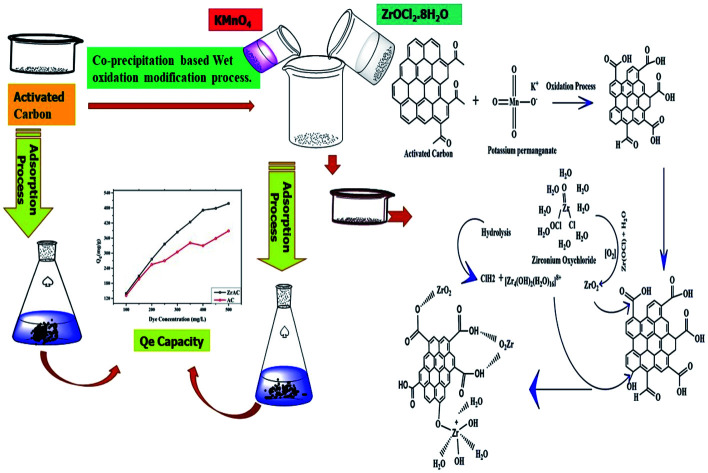

## 1. Introduction

Activated carbon (AC) is a processed form of carbon-based materials consisting of microscale pores with high surface area, broad surface reactivity, diverse pore size distribution and chemical as well as thermal stability.^1^ The activated carbons have been amply used in various separation techniques of solid–liquid interface pollutants, gaseous pollutants, pharmaceutical waste, catalytic materials, and elimination of industrials pollutants (inorganic or organic).^2^ However, for adsorption purposes, it has been extensively used for years in different forms such as granular, powdered, tubes, composites, *etc.* with or without modifications.^3,4^ For the adsorption process, despite varied microporosity, the specific interaction between adsorbate and adsorbent also plays a vital role in the adsorption process.^5^ Sometimes these interactions do not prove to be effective for macromolecules, or other complexed molecules. Coincidentally, the activated carbon faces the same interaction difficulties for complex molecules that constrains its application for different macro-structured pollutants.

Moreover, some other limitations of AC such as prolonged equilibrium period, tedious regeneration capabilities *etc.* obstructs the direct application of AC.^1^ Typical surface characteristics of AC assists in the modification process that makes them more feasible for any targeted pollutants. So nowadays, researchers have been focussing more on the preparation of highly compatible materials that are more specific to the pollutant.^6,7^ Researchers have already reported that the modification of activated carbon, superimposed crucial and significant impact over the removal of targeted contaminants.^5^

Improvement in the adsorptive capacity of activated carbon has been achieved either by different surface functional groups modification or by impregnation of metals with appropriate chemical or physical treatments or both. Metal impregnation or oxidative functionalization has been carried out, after the activation process by treating with appropriate chemicals in liquid or gaseous phases.^8,9^ These impregnation and oxidation processes have a higher influence on the modification of AC, as it enhances the number of active sites of the surface. Ion exchange, wet oxidation process, or chemical deposition followed by calcination/reduction step, *etc.* are the known metal impregnation methods.^10,11^ Besides this, researchers have also reported that most of the activated carbon comprises mainly of microporous structures that are not uniformly present over the surface. These pores although plays an essential role in the adsorption of smaller molecules but for larger molecules, diffusion and pore blockage, become the major limiting factors.^12,13^ To overshadow this issue, the modification should be in a way to develop pores of various sizes. Some researchers reported that the mesoporous carbon possesses enlarged porosity, thermal stability and electrical conductivity that plays a pivotal role for adsorption, catalysis and other processes.^12,14^ Usually, impregnation has been carried out using different transition metals and organometallic compounds of various elements such as Al, Zn, Fe, Ag, and Ti.^15–17^ The utility of different transition metal is well-known in the field of water treatment as these metals do not leach out into water owing to their high positive character.^18^ Among different transition elements, zirconium is more superior over other elements in terms of efficiency, cost and toxicity. Literature also suggested the zirconium(iv) application, in the form of metal salts, does not exhibit any toxic effect on the aquatic environment.^19–21^ Moreover, zirconium impregnation in its hydrated form makes the adsorbents an anion exchanger that promotes more removal of the anionic counterpart.^22^ Zirconium also exhibits highly electropositive character responsible for a strong affinity towards the electronegative ions, thus also show strong affinity towards the anionic dye.

Reactive dyes are a well-known anionic dye that is used extensively in dye industries because of their easy dyeing application, availability and vibrant colours.^19^ These dyes are one of the most thrust areas for environmental concerns as they are the top polluters of the environment. These dyes generally released from dyeing and printing units of textiles that possess toxic effluent. Being more soluble with water, its concentration in effluent discharge increases as compared to other groups of dyes. Moreover, their recalcitrant and toxic nature and their high oxygen demand,^23^ makes them more potent to be removed from the environment using reliable and competent techniques. Adsorption is one of the simplest treatment processes for the removal of dyes wastewater. Some researchers had reported the use of different metal-doped over activated carbon for the removal of different classes of dye, but their adsorption capacity makes them incompatible.^24^ So, grabbing the advantages of zirconium(iv), the present study mainly devoted on the impregnation of zirconium on AC for the removal of an anionic reactive dye, named Reactive Blue 19 (RB19).

Hence the present study mainly focuses on the pioneer use of zirconium oxychloride salt as an impregnated material on activated carbon to develop a novel adsorbent for the removal of anionic reactive dye RB19. The studies targeted to develop a novel material that is stable, non-toxic, efficient and eco-friendly. The study aimed to synthesise material having high surface oxidative functionality, diverse porosity and thermally stable mesoporous material using simple modified wet oxidation process, that can potentially target reactive dye. The studies also examined and compared all the characteristic features of the novel developed material and raw AC using the different instrumentational tool. FE-SEM, EDX, TGA, BET, XRD and XPS has been used to investigate all the morphological, textural and surface functional features of the novel material. Finally, a series of batch adsorption experiments, isothermal behaviour, rate of adsorption and thermodynamic parameters along with regeneration studies have also been conducted to understand the applicability and feasibility of the developed material.

## 2. Materials and methods

### 2.1. Materials and reagents required

Activated carbon was procured from R & M Chemicals, zirconium oxychloride (ZrOCl_2_·8H_2_O) from Loba Chemicals, while potassium permanganate (KMnO_4_), and sodium bicarbonate (NaHCO_3_) were purchased from Remkem Chemical Ltd. The modelled dye, *i.e.* reactive blue 19 was purchased from Sigma Aldrich. All chemicals were of analytical grade and used without any further treatment.

### 2.2. Preparation of the zirconium impregnated activated carbon

The modification of activated carbon was carried out using a wet oxidation process and restructured as per the requirement of the study. For this, 10 g of raw activated carbon was pre-heated for 3 h in a hot air oven at 120 °C for proper activation and drying. After this, the AC was cooled and mixed with 1 M KMnO_4_ solution with continuous stirring on a magnetic stirrer for about half an hour at 250–270 rpm for oxidizing the surface of AC. Then, the mixture was diluted with Millipore water and filtered. The filtered solid residue was again mixed with 1 M ZrOCl_2_·8H_2_O at 220 rpm for about 10 hours. The final suspension was filtered and washed with 1% NaHCO_3_ solution and immersed overnight in the same. Subsequently, the suspension was decanted and washed with Millipore water until the pH stabilized close to neutral. Lastly, the filtered residue was air-dried for 4 h and then kept into hot air oven for complete drying. The final dried solid was stored in a desiccator, to avoid moisture regain for later use and finally referred to as ZrAC.

### 2.3. Materials characterization

The raw activated carbon and modified activated carbon were analyzed by using the different textural, structural and compositional instrumental methods. The spectral surface morphology and elemental properties were examined by using Field Emission Scanning Electron Microscope (Supra 55 Carl Zeiss, Germany), equipped with Energy Dispersive Microanalysis (Oxford Liquid Nitrogen free SDD X MAX 50 EDS). For this samples were finely ground and dried entirely before mounting over carbon sticky stub. The platinum coating was performed on both adsorbents to get high-quality images.

The textural characteristics of both the samples were calculated by using nitrogen (N_2_) adsorption–desorption isotherms at 77 K using Autosorb-1C, Quanta chrome, USA. The surface areas, porosity and pore size distributions were estimated by BET isotherms and DFT (density functional theory) model with the help of software equipped with the instrument that enables the user to examine the pore size along with pore volume and surface area. The samples were degassed prior to the detailed analysis, to avoid inaccuracy in the results.

Later, the presence of different functional groups was analyzed by identifying the changes in vibrational frequencies in the functional groups with the help of FTIR spectrophotometer (PerkinElmer). The samples were analyzed by preparing KBr pellets by mixing both adsorbents with KBr powder in a definite ratio to form a thin disc. The data were recorded from 4000–400 cm^−1^ at 0.4 cm^−1^ resolution. The influence of CO_2_ and water was always subtracted during the recording of data. Further, the crystalline structure of both the adsorbents was analyzed by using Bruker-D8-Advance X-ray diffractometer at a voltage of 40 kV and current of 40 mA, in the range of diffracting angle 2*θ* from 5° to 120° at a step size 0.05° using 2.2 kW Cu Kα as X-rays source and LINXEYE XE detector.

The thermal characteristics of AC and ZrAC were investigated using thermogravimetric analysis (TGA) (EXSTAR-Model SII 6300 EXSTAR, Canada) from 30 to 900 °C with a ramping rate of 10 °C per minute under nitrogen environment at a flow rate of 200 mL min^−1^. XPS measurement was also conducted to study the detailed elemental composition as well as different oxidation states of the surface element. For this, the X-ray photoelectron spectrophotometer (Physical Electronics Model-PHI 5000 Versa Probe III, Japan) was used having a monochromatic lamp with an average analysis depth of approximately 5 nm.

### 2.4. Batch adsorption experiments

Preliminary adsorption experiments were carried out to investigate the potential of the ZrAC as compared to AC. The stock solution of the dye was prepared and diluted accordingly as per requirement using Millipore water. Before initiating the batch experiments, a trial set of experiments has been carried out to optimized the pH, dose and agitation time and speed of the respective adsorbents. 100 mL of the dye solution with varying concentrations (100 to 500 mg L^−1^) were agitated in an Erlenmeyer flask (250 mL capacity) with an adsorbent dose of 70 mg at a speed of 120 rpm for 90 minutes. The initial and final concentration of the dye solution was determined by using UV-Vis spectrophotometer (LabTech Model 9100A) at the maximum wavelength (*λ*_max_ = 595 nm) of the dye and their concentrations were calculated with the help of the standard curve. The removal percentage was calculated by simple mathematical calculation and maximum adsorbent capacity was calculated using eqn (1).1
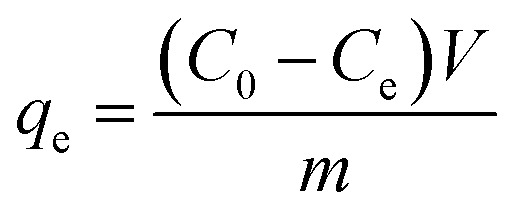
where, *C*_0_ and *C*_e_ represent the initial and equilibrium concentration (mg L^−1^) of the dye solution respectively, *V* (L) represents the total volume of dye solution used in each experimental run and *m* (g) represents the dry mass of the adsorbent used for the adsorption process.

The equilibrium data have been further investigated by isotherms studies, that helps in describing the relationship between the liquid phase (adsorbate concentration) and the solid phase (adsorbent) at constant temperature and helps in designing the adsorption systems.^25^ In the present study, non-linear two parametric isotherms, *i.e.* Langmuir and Freundlich, were applied by using eqn (2) and (3), respectively, to determine the fitting behaviour of the sorbents. Langmuir describes the monolayer adsorption behaviour of the adsorbate onto the surface of the adsorbent, containing finite adsorption sites with no transmigration of adsorbate molecules in the surface plane.^26^ In contrast, Freundlich isotherm,^24^ assumes non-uniform surface energies that used to describe the equilibrium features of the adsorbate for the heterogeneous surface.

Non-linear form of Langmuir isotherm is represented as:2
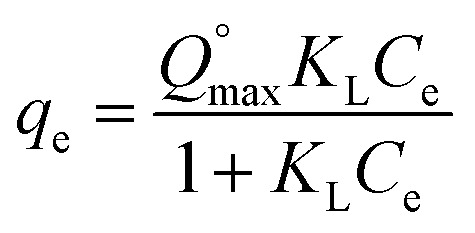


Non-linear form of Freundlich isotherm is expressed as:

3 *q*_e_ = *K*_F_*C*_e_^*n*^

where, 
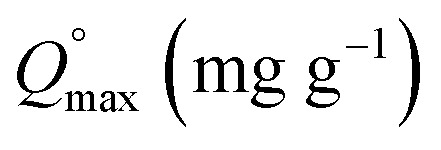
 is maximum monolayer saturation capacity of the sorbent, *C*_e_ (mg L^−1^) is a concentration of adsorbate at equilibrium condition, *q*_e_ (mg g^−1^) is the amount of adsorbate uptake at equilibrium and *K*_L_ (L mg^−1^) is Langmuir constant. *K*_F_ (mg L^−1^)^*n*^ represents the Freundlich constant and *n* is the dimensionless Freundlich intensity parameter, that defines the magnitude of adsorption driving force. In case of best fitting of experimental data with monolayer adsorption mechanism, then the model can also be described by a dimensionless constant named as equilibrium parameter or separation factor,^27^ represented as *R*_L_ (eqn (4)). This factor defines the isotherm shapes as if *R*_L_ = 0; *R*_L_ < 1; *R*_L_ = 1 & *R*_L_ > 1, infers irreversible, favourable, linear or unfavourable isotherm respectively.4
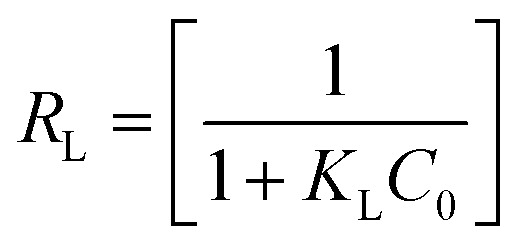
where, *R*_L_ represents separation factor (constant), *K*_L_ represents the Langmuir constant and *C*_0_ (mg L^−1^) is the initial adsorbate concentration.

Another isotherm, name Sips isotherm was also used to investigate the adsorption behaviour of the sorption process of both the adsorbent.^28^ The Sips isotherm, in general exhibit the combined form of Langmuir and Freundlich isotherm expression that are deduced for predicting the heterogeneous adsorption system.^28^ The model is expressed as follows:5
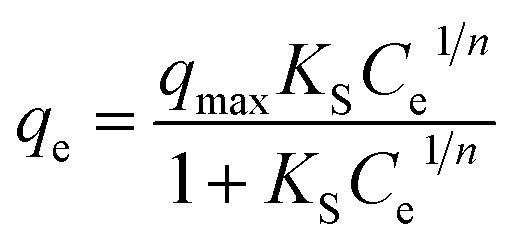
where, *q*_max_ (mg g^−1^) represents the predicted maximum adsorption capacity of the adsorbent, *n* is heterogeneity factor and *K*_S_ (L mg^−1^) represents Sips constant.

### 2.5. Dye adsorption kinetics and thermodynamic study

Three kinetics models, *i.e.* pseudo-first-order, pseudo-second-order and intra particle diffusion model, were used in this work to predict the rate of sorption.

#### 2.5.1. Pseudo-first order

The linearized form of the model was developed by Lagergren^29^ and expressed as follows:

6 ln(*q*_e_ − *q*_*t*_) = ln *q*_e_ − *kt*

where, *q*_e_ (mg g^−1^) and *q*_*t*_ (mg g^−1^) represents the adsorbent adsorbed the dye molecules at equilibrium and at any time, *t* respectively. *k* (min^−1^) represents the pseudo-first-order kinetics. The values of *k* and *q*_e_ have been calculated from slope and intercept of the graph plotted ln(*q*_e_ − *q*_*t*_) *versus t* respectively.

#### 2.5.2. Pseudo-second order

The adsorption equilibrium can also be described as a pseudo-second-order model, assuming that the adsorption rate of encapsulated sites of adsorbent is proportional to the number of vacant sites.^25^ The equation based on equilibrium adsorption can be expressed as:7
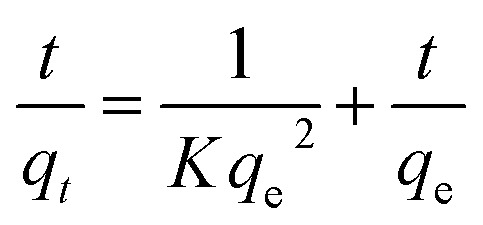
where, *K* (g mg^−1^ min^−1^) is the pseudo-second-order rate constant, *q*_*t*_ and *q*_e_ are same as in pseudo-first-order. The values of *q*_e_ and *K* has been calculated from the slope and intercept of plot *t*/*q*_*t*_*versus* time, respectively. The pseudo second order rate constant (*K*) was also used to calculate the initial rate of sorption by following eqn (8).

8 *h* = *Kq*_e_^2^

#### 2.5.3. Intra particle diffusion model

The intraparticle diffusion model was proposed by Weber and Morris^30^ and was used to identify the adsorption mechanism of any adsorbate onto the adsorbent. The equation is expressed as:

9 *q*_*t*_ = *k*_i_*t*^1/2^ + *C*

where, *k*_i_ (mg g^−1^ min^−1/2^) represents the intraparticle diffusion model's initial rate constant, *C* (mg g^−1^) is the intercept representing a constant related with the thickness of the boundary layer. Also, a higher value of *C* can directly be correlated to a greater effect on the limiting boundary layer. This model also helps in identifying the reaction pathways and revealing the adsorption mechanism along with predicting the rate-controlling step of the adsorption mechanism.

#### 2.5.4. Thermodynamic study

The thermodynamic behaviour of the adsorption can be described by calculating the standard parameters such as Gibbs free energy (Δ*G*°, kJ mol^−1^), enthalpy change (Δ*H*°, kJ mol^−1^) and entropy change (Δ*S*°, kJ mol^−1^) by using following equations.^25^

10 Δ*G*° = −*RT* ln *K*_c_ (Van't Hoff equation)

11
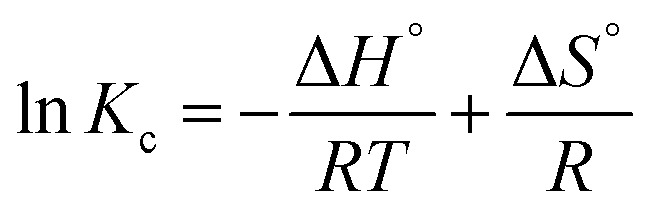
where, *R* is a universal gas constant of value 8.314 J mol^−1^ K^−1^, *T* is the absolute temperature in kelvin and *K*_c_ represents the unitless distribution coefficient, which can be derived as12
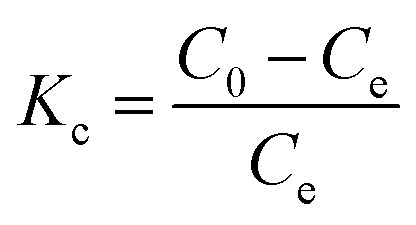
where, *C*_0_ and *C*_e_ represent the initial and equilibrium RB19 dye concentration, respectively.

The values of Gibbs free energy (Δ*G*°) can be derived from Van't Hoff equation and the values of Δ*H*° and Δ*S*° were calculated from slope and intercept of the linear regression plot between ln *K*_c_*versus* 1/*T*, respectively.

### 2.6. Regeneration and desorption studies

The regeneration of the utilized adsorbent has been investigated to evaluate the practical applications and economic feasibility of the adsorption process. The number of eluents of different nature has been investigated for desorption studies. For this one acidic (0.1 M H_2_SO_4_), one alkaline (0.1 M NaOH), one salt (0.1 M NaCl) and distilled water have been used as desorbing agents. Initially, the adsorption process has been carried out with 0.1 g of AC and ZrAC to treat 100 mg L^−1^ of dye concentration for 90 min (equilibrium time). Then, the loaded adsorbents were separated from the adsorbate solution and again agitated with the eluents at the same reaction time, for desorption study. The desorption percentage was calculated from the following equation13
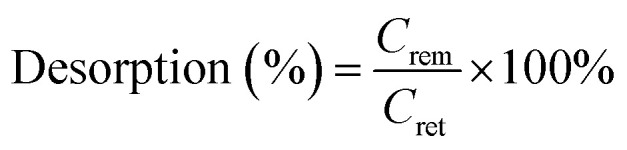
where, *C*_rem_ and *C*_ret_ refer to the amount of dye removed and the amount of dye molecules retained on the exhausted adsorbent.

After each cycle of treatment, the adsorbent was washed and soaked into distilled water and after filtration, the solid residue was again used for next cycle treatment. This process of adsorption–desorption process has been carried out for five cycles, as at each cycle, small loss of adsorbent will possibly occur.

## 3. Results and discussion

### 3.1. Synthesis of zirconium impregnated AC

**Fig. 1:**
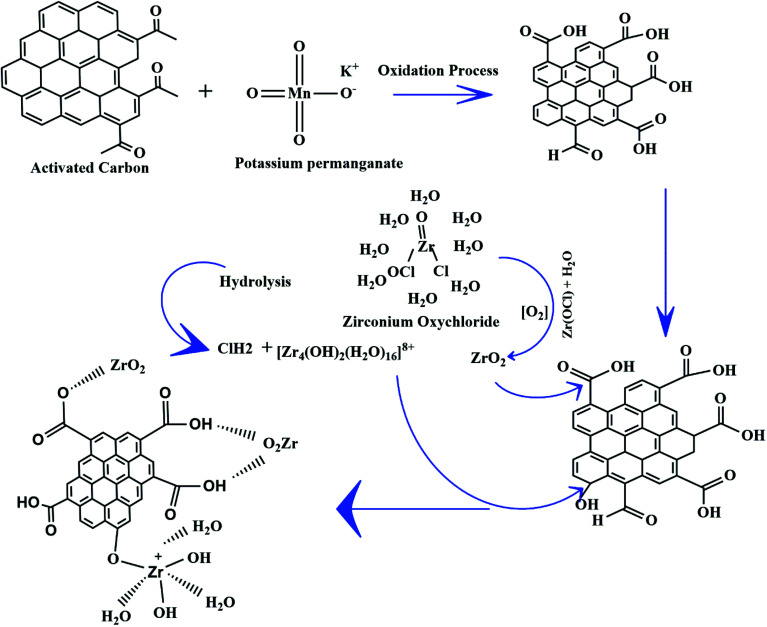
Schematic representation of possible modification process takes place while ZrAC synthesis.

The modification process has been commenced over raw activated carbon to ensure the surface must have a cationic counterpart that will attack the targeted anionic pollutant. For this, a defined methodology has been adapted to develop new material having higher adsorption affinity for the anionic dyes. The probable reaction mechanism of the developed material has been given in Fig. 1. Firstly, the surface of the activated carbon has been oxidised using potassium permanganate solution so that the surface of activated carbon increases its affinity towards metal counterpart. On reaction with KMnO_4_, the carbonyl group present on the surface of the activated carbon gets oxidised into a carboxylic group that further readily reacts with the zirconium oxychloride. On hydrolysis, zirconium oxychloride dissociates into a dimmer form, [Zr_4_(OH)_2_(H_2_O)_16_]^8+^ along with HCl and some oxychloride forms. The oxychloride forms further get oxidises to zirconium oxides (ZrO_2_) while dimmer form reacts with AC and forms complex. Lastly, the formed solid was treated with sodium bicarbonate solution, that being amphoteric, readily reacts with the remaining acidic groups to prevents further complex formation. These modification processes were further validated with different characterization techniques that ensures the formation of metal oxides on the surface of activated carbon.

### 3.2. Characterization techniques validation

#### 3.2.1. Microscopic studies of the activated carbon and modified activated carbon (ZrAC)

**Fig. 2:**
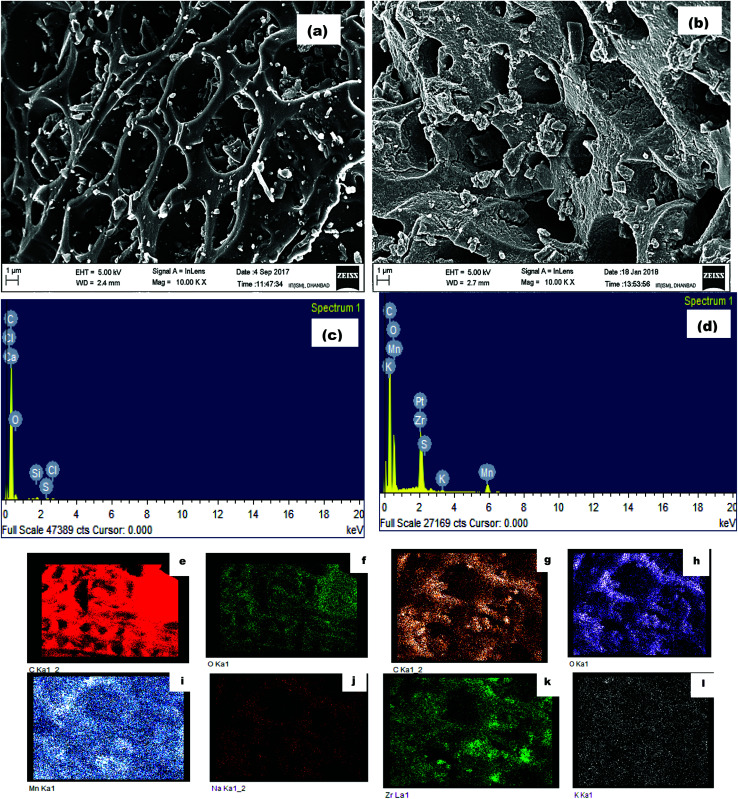
Microscopic morphology of (a) AC and (b) ZrAC, EDX spectra of (c) raw and (d) ZrAC and elemental mapping of AC (e and f) and ZrAC (g–l).

To study the morphological changes of AC and ZrAC, FE-SEM of both the materials, along with EDX have been carried out and the results are shown in Fig. 2. The same resolution images were preferred. The results depicted distinguish morphology of both the materials as compared together. The raw AC has a homogeneous surface structure with voids whilst, the surface of modified carbon (ZrAC) exhibited highly corroded and irregular structure all over the surface, that possibly because of chemical treatment it goes through. The metals impregnation process oxidises the surface through complex formation or by reacting with hydrogen bond, that results in residing of metals within the pores or at the outer boundary of the pores.^14,31^ The FE-SEM images demonstrated the same; the metals distribution was not uniform throughout and are either present inside the pores or at the boundary of the pores, resulting in the widening of the pores. This changes of the surface morphology of newly developed materials from smooth and regular pores size to rough and irregular surface structure with wider pores occurs, because of the formation of new different active sites on the carbon structure. Also, the surface of modified carbon possessed extended porosity that may be due to the entrenchment of the Zr metals into the pores and their complex formation with the active sites of available oxygen.

Concomitantly, the EDX results also affirmed that the oxidation process with KMnO_4_ and zirconium oxychloride remarkably increases the oxygen moieties on the ZrAC. The elemental composition results presented in ESI Table S1† claimed the impregnation of Zr metals over the carbon surface with 14% presence of Zr elemental composition. The presence of other oxidizing elements, *i.e.* oxygen and manganese also confirmed the oxidizing activity inbuilt in case of ZrAC. During the modification processes, the oxygen moieties become more than double as compared with raw activated carbon that shows a good sign for the application part. Thus, the results of FE-SEM and EDX mapping conjointly revealed that the modification changes the structural pattern of the raw AC into the more roughed oxygen-containing surface with diverse pores, that will help into its application.

#### 3.2.2. Textural properties analysis

**Fig. 3:**
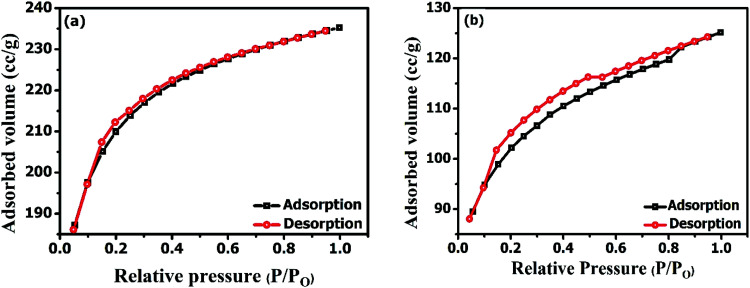
The plots depicted the nitrogen (N_2_) adsorption–desorption curve of (a) AC and (b) ZrAC.

To understand the physical properties of the AC and ZrAC, the nitrogen (N_2_) adsorption–desorption, pore size distribution and pore volume were analyzed using BET, BJH and DFT model respectively. The Fig. 3(a and b) represented the adsorption branch of both the material exhibited the type I isotherms according to IUPAC classification that revealed the presence of microporous characteristics of the materials. But the sharp increase of N_2_ uptake at very low pressures in case of AC revealed that the existence of more micropores than ZrAC.^32^ The presence of more developed hysteresis loops in modified carbon exhibits the capillary condensation of pores filling by a secondary process and accredited to the development of mesopores in ZrAC,^33^ that helps in adsorption of larger molecules. The type of hysteresis loop present in ZrAC might represents either hybrid type of H1 and H2 (a) hysteresis loop that further exhibited the presence of narrower range of uniform mesopores with pore-blocking phenomenon^33^ or may exhibited bimodal porosity, resulting in step-wise porosity that overview the fact that at lower pressures, the encapsulated mesopores emptied than the open pore of the same sizes.^34^ Moreover, the pronounced hysteresis loop at higher *P*/*P*_0_ in ZrAC (above 0.45), also affirmed the formation of mesoporosity as suggested by other authors.^34,35^ A similar transformation of micropores to mesopores and macropores has also been reported by some other scientists,^35,36^ after impregnation with the metals.

The graphical instances were further validated with the calculated porosity parameters data from N_2_ adsorption–desorption isotherms and depicted in Table 1. It was observed that the specific surface area of AC had been more than 500 m^2^ g^−1^ resembling the presence of the more micro porosity with narrow pores while the small specific surface area of ZrAC showed characteristics for meso or macro porous solid. It has been seen that the relative micro porosities (*V*_micro_/*V*_t_) and the micropore volume of the ZrAC get reduced (approximately 50%) suggesting the filling of pores with the metal.^36^ Also, the mean pore diameter for ZrAC was found 2.14 nm implying that width of the pores gets enlarged with the impregnation of the Zr particles illustrating deposition of the particles around the pores. The range of pore width clearly represents the mesopores region as per the IUPAC classification.^37,38^ The results of the specific surface area and the mean pore width are in reverse relationship suggesting a good agreement between them. The contribution of micropore volume to the total pore volume also reduced to affirm the modification has occurred into the micropores area. Microporosity has been changed to meso as well as macro porosity for the extended applications.

**Table 1:** Surface area, pore size and pore volume of the activated carbon and modified activated carbon^a^

Samples	*S*_BET_ (m^2^ g^−1^)	*S*_micro_ (m^2^ g^−1^)	*S*_ext_ (m^2^ g^−1^)	*V*_micro_ (cm^3^ g^−1^)	*V*_t_ (cm^3^ g^−1^)	*V*_ext_ (cm^3^ g^−1^)	*D*_p_ (nm)	*V*_micro_/*V*_t_
AC	750.9	712.4	38.51	0.3139	0.3648	0.8158	1.943	0.86
ZrAC	363.2	331.3	31.89	0.1500	0.1941	0.0441	2.138	0.77

a *S*_BET_ – BET surface area; *V*_t_ – total pore volume; *V*_micro_ – micro-pore volume; *D*_p_ – BJH adsorption mean pore width.

#### 3.2.3. Thermogravimetric analysis of AC and ZrAC

**Fig. 4:**
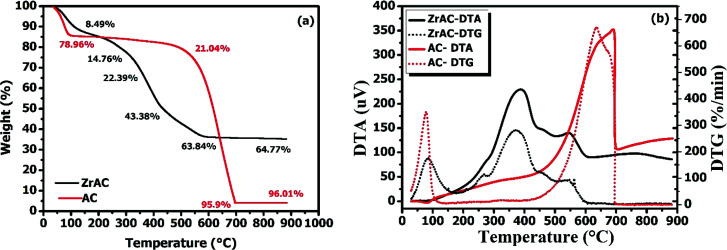
The graph depicted thermal characteristics of both the materials (a) using TGA and (b) DTA–DTG methods.

For understanding the different properties of the materials with a change in temperature, the thermogravimetric study has been commenced and presented in Fig. 4. It has been found that the total mass loss of ZrAC was 64.77% having maximum mass loss rate (*T*_max_) at around 550 °C while the raw AC found to be degraded up to 96.01% of total mass with *T*_max_ 600 °C, representing comparatively less stability of ZrAC. In ZrAC, the initial weight loss around 100–150 °C of about 10–12% that attributed to the loss of volatile compounds and absorbed water present on the surface of the adsorbent. This result was further validated for the above steady-state condition by heat-treated ZrAC (Fig. S1†). Further, the weight loss around 200 °C may be assigned because of removal of oxygen-containing functional groups^39^ that later extended to the weight loss of about 63.84% up to 600 °C temperature, corresponding to further decomposition of different functional group (especially carboxylic groups). Similar trends were seen in heat-treated ZrAC.

Later, the DTA graphs manifested the modification process over activated carbon by indicating a broad endothermic peak around 100–150 °C, representing the desorption of water molecules, followed by one sharp exothermic peak at around 400 °C that starts around 300 °C attaining its peak at 400 °C and gradually declines at about 450 °C, suggesting dehydroxylation of the material.^40,41^ Later one small exothermic was also observed in between 500-585 °C followed by one endothermic peak at around 600 °C that represents a gradual decrease of the base carbon material. Additionally, the results were further assured by the DTG curve that exhibited one small peak in between 100–180 °C followed by one small peak in between 300 °C followed by on sharp peak at 400 °C and a small peak in between 500 to 600 °C, that ascribed the multistep decomposition behaviour of the material. Likewise, in AC a slightly more weight loss of around 15% at 100 °C was observed, because of dehydration of water, indicating high absorbed water in micropores corresponds to high hydrophilicity of the activated carbon surface. This is followed by a gradual decrease in weight up to 41.8% till 600 °C and then final total loss of weight of about 96.01% at around 885 °C indicating gradual degradation of carbon base. Simultaneously, DTA also supported the TGA by showing exothermic peaks at 690 °C only while DTG showed one peak at 634 °C and one small peak at 677 °C representing slow degradation of carbon base material of AC. The temperature degradation of both the samples illustrated that the thermal stability of ZrAC was not enhanced, but enough stable for water treatment^42^ and their degradation temperature difference also revealed that the ZrAC content 31.44% of the metal counterpart that affirmed the modification was achieved. Thus, a successful transformation of the pristine activated carbon has been achieved by metal impregnation using a wet oxidation process.

#### 3.2.4. FTIR interpretation

**Fig. 5:**
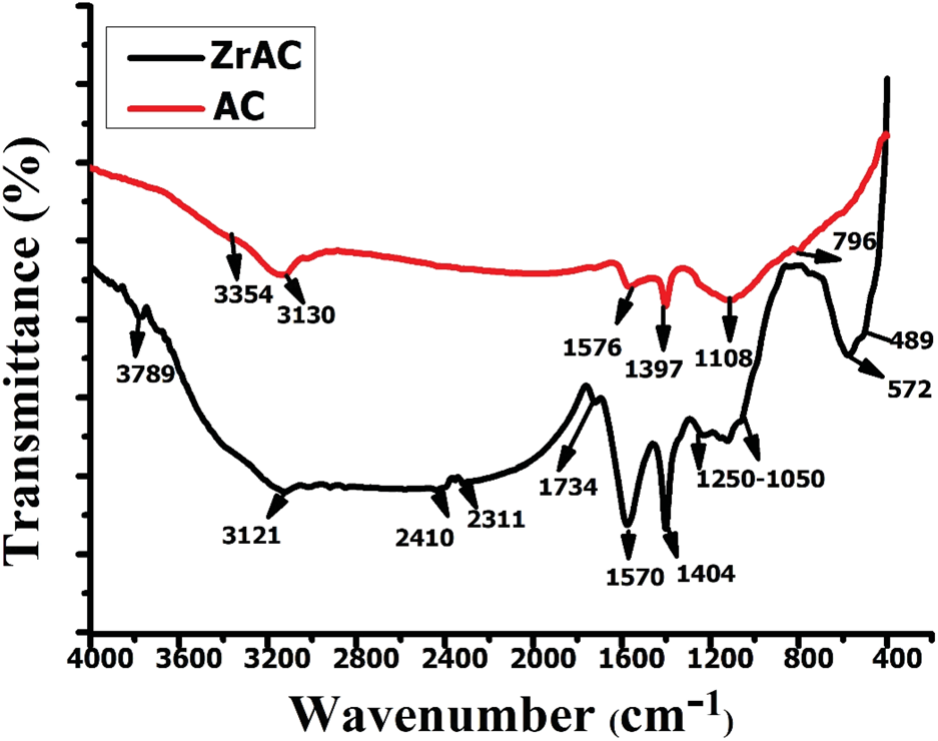
The FTIR spectral curves of AC and ZrAC.

The FTIR spectral results were analyzed and represented in Fig. 5. The analysis of spectral curves clearly showed the presence of more peaks in modified activated carbon because of the participation of more functional ions that leads to the addition of more functionality. The presence of peaks in between 4000–3200 cm^−1^, in ZrAC curve, represents the O–H stretching vibration in carboxyl and phenol groups,^43^ while in raw AC peak at 3354 cm^−1^ corresponds to the adsorbed water due to stretching and bending modes of water. The peaks between 3100–3000 cm^−1^ are assigned for C–H stretching present mainly in unsaturated compounds and both samples have the same carbon stretching. The weaker peak at 3121 cm^−1^ in ZrAC and stronger peak at 3130 cm^−1^ in raw AC revealed that the modification process changes the carbon stretching. The presence of peaks at 2410 cm^−1^ and 2312 cm^−1^ in ZrAC represented either the alkynes stretching with elements such as N, O, S or the presence of atmospheric CO_2_ that the material has adsorbed.^36^ The IR spectra of ZrAC showed strong peaks in between 1870–1550 cm^−1^ that are attributed to C=O group of esters, ketones, amides, carboxylic acids, their salts or may acid anhydrides, disclosing more oxidising functional groups addition after the modification. The presence of one more sharp peak in ZrAC at 1570 cm^−1^ confirms the presence of C=O stretching corresponds to carbonyl group present in aldehydes and ketones^39^ while another sharp peak centered at 1404 cm^−1^ showed the presence of C–O–C vibrations of epoxy or alkoxy groups.^42,44^ Furthermore, the peaks at 1249–1050 cm^−1^, 1088 cm^−1^ were assigned for C–O stretching of carbonyl groups and Zr=O respectively. Additionally, one small peak at 572 cm^−1^ represents the presence of organo-halogens, that attributed to Cl^−^ ions get attached to the carbon skeleton,^45^ whilst one small peak was also found at 487 cm^−1^ that assigned the presence of Zr(iv), in monoclinal and tetragonal phase of ZrO_2_ ^40^ in the modified sample. Hence, the excerpt of the FTIR results clearly indicated that the ZrAC comprises of more reactive oxidative functionalities as compared to the native AC that will readily react with the dye molecules. Furthermore, the FTIR spectra of the AC and ZrAC after adsorption of dye molecules were also examined and presented in Fig. S2.† The spectra show distinguished peaks showing the interaction of dye molecules with the respective adsorbent. In ZrAC-post, the vibrational peaks around 3100–3000 cm^−1^ were observed representing –OH stretching vibrations while a sharp peak develops at 3137 cm^−1^ in AC-post. After adsorption, the sharpness of peaks at 2350 cm^−1^ and 2330 cm^−1^ in AC and ZrAC respectively, was developed due to alkyl group that interacts with the adsorbents. In ZrAC-post adsorption, a sharp and broad peak at 1569 cm^−1^ with slight shifting, were observed showing zirconium ions gets interacted with the amine group of dyes molecules.^46^ Also, at 1168 cm^−1^ a sharp peak in ZrAC after adsorption is observed resembling the interaction between –OH group with Zr^4+^ ions.^47^ The peaks at the region 1500–1600 cm^−1^ in both the adsorbent indicate the pi–pi interaction of reactive dye molecules with the surface of activated carbon.^48^ The appearance of vibrational shifted peaks at around 900–700 cm^−1^ in ZrAC after adsorption, ascribed the metal–oxygen interactions.^14^

#### 3.2.5. X-ray diffraction (XRD) analysis of AC and ZrAC

**Fig. 6:**
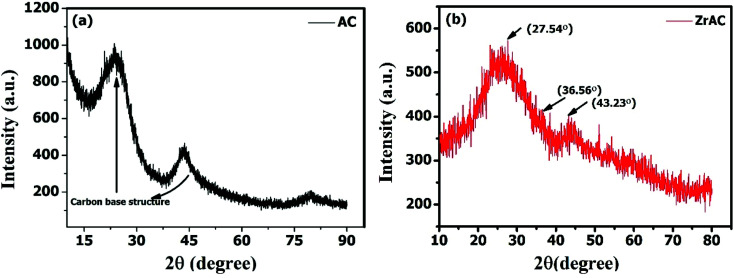
X-ray diffraction spectra of activated carbon and modified activated carbon (ZrAC).

The powdered X-ray diffractogram has been carried out for AC and ZrAC in the 2*θ* range of 10° to 90°, to identify the nature of the materials as well as a polymorphic form of zirconium in ZrAC. Undoubtedly, the graph (Fig. 6) showed the amorphous nature of the material as a major portion of the material constitute of carbon substrate base. The raw AC has two prominent peaks at 2*θ* of around 25° and 44.5° corresponds to the plane (002) and (101) respectively, resembles the graphitic hexagonal structure of the carbon-based material.^49,50^

Unlike the raw AC, in ZrAC, it was found that the graphitic peaks were shifted and the carbon strand was distorted, because of the encapsulation of Zr(iv) over AC. It has also been observed that the treatment interaction between the metal salt and activated carbon, interfered the graphitic structure leading to its disrupt structure. Similar results of distortion were observed by some other scientists after impregnation of zirconium metals.^36,51^ Besides this, some few peaks are found to appear in the XRD graph of ZrAC but are not sharp and stronger one. This lack of specific prominent peaks is because of the higher concentration and phase purity of carbon-based material that causes dominance over metal concentration.^14^ However, the graph shows the XRD patterns of ZrAC, where two small peaks at 2*θ* angles of 27.54° and 43.23° that may attribute to monoclinic phase while at 36.56° corresponds tetragonal structure of zirconia,^36,47^ as zirconia exist in three polymorphic phases, *i.e.* monoclinic, tetragonal and cubic, at ambient temperature. These peaks were further affirmed by conducting heat treatment of the material so that the carbon material has been burn out and their interference would be minimised. The subsequent XRD results of the heat-treated material have been assessed and it was observed that the developed peaks were clear and had been recognized for polymorphic phases of zirconium (Fig. S3†) suggesting the monoclinal and tetragonal phase of the metal. Hence it was finally observed that the modification process had changed the structure of the carbon material with some new crystalline structure.

#### 3.2.6. XPS analysis of ZrAC and raw AC

**Fig. 7:**
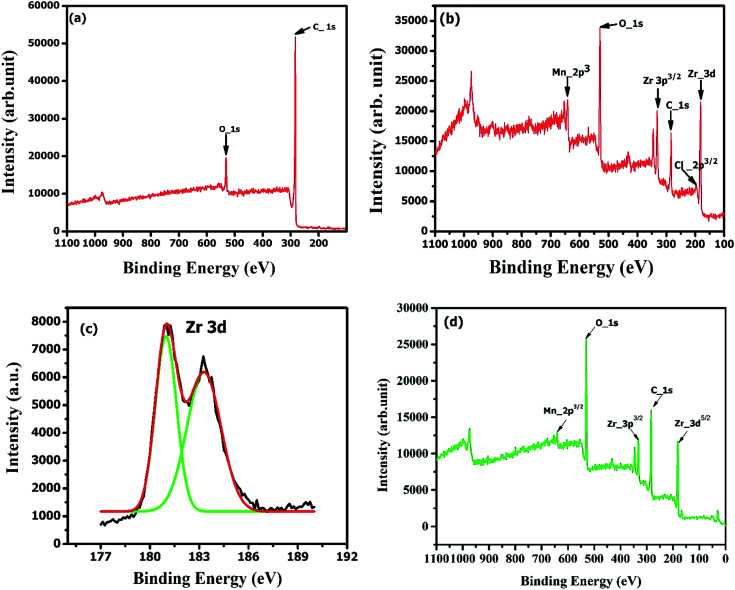
XPS full spectra plots of (a) raw AC, (b) ZrAC along with (c) deconvoluted peaks representing the Zr peaks separately and (d) ZrAC-post adsorption.

The XPS analyses were carried out on representative samples to identify the detailed surface functionality and possible structural form of Zr(iv) in the modified carbon after the impregnation process. Fig. 7 represents that on impregnation, the elemental composition has been changed. The raw activated carbon has two prominent peaks [Fig. 7(a)] representing the carbon and oxygen group, while ZrAC possesses more oxidizing elements. The two peaks of manganese at 641.6 eV and 652.9 eV represent 2p^3/2^ and 2p^1/2^ state, respectively of the element showing its catalytic and oxidative properties.^52^ Similarly, the 3d region of Zr metal [Fig. 7(b) and (c)] at the binding energies of 332.5 eV and 181.5 eV corresponds to Zr(iv) in ZrO_2_.^39^ The zirconium metal was observed in two forms, *i.e.* 3p^3/2^ and 3d^5/2^ representing the oxidizing nature of the metal. Again, a small peak of Cl 2p was observed for ZrAC with a binding energy of 198.6 eV, which agreement completely with ZrAC.^53^

Besides these, the peak intensities of carbon C 1s and O 1s in both the materials were observed in the range of 283–285 eV and 529–531 eV respectively. There was a slight shifting of the binding energies after impregnation of the metal which is in good agreement with the NIST database.^36^ Consequently, it was also observed that the oxygen surface compositions are higher in ZrAC than AC and *vice versa* for the case of carbon surface composition, affirming more oxidizing surface and a high degree of functionalization on ZrAC as compared to raw activated carbon.^36^ This interference was further confirmed by the atomic concentration (atomic weight%) presented in Table 2, that revealed the change in the composition of the activated carbon upon impregnation with the metal. A remarkable elevation of oxygen composition has been seen in ZrAC, due to the high degree of oxygen moieties in oxidizing agents as well as in zirconium oxychloride.

**Table 2:** Summary of atomic surface composition of XPS analysis of ZrAC and AC

Elements	C 1s	O 1s	Mn 2p^3^	Zr 3d^3^	S 2s	Cl 2p	Na 1s	K 2p	S 2p
Atomic (%) AC	89.84	8.84	—	—	—	—	0.39	—	0.93
Atomic (%) of ZrAC	34.7	46.6	5.5	10.4	<0.1	1.0	1.8	<0.1	<0.1
Atomic (%) of ZrAC-post adsorption	54.86	31.13	3.59	6.30	0.99	3.13	—	—	—

Contrary to this, the carbon composition of ZrAC was too low as compared to pristine activated carbon. Thus, the refined spectral analysis results by XPS, thereby concomitanted the EDX results suggesting the successful impregnation and enhancement of oxidizing surface of modified carbon using wet oxidation process for future use as an adsorbent. Further, the XPS spectra of ZrAC-post adsorption [Fig. 7(d)] resembles binding energy of 331.61 eV and 180.53 eV in the Zr 3d region with an agreement with the formation of ZrO_2_.^37,54^

### 3.3. Sorption performance

#### 3.3.1. Effect of initial pH of the dye solution

The working solution pH plays a significant role in the adsorption process as it influences both the surface charge of the adsorbent and structure of dye molecules. The pH also affects the degree of ionisation of functional groups that adsorbents carry. In the present study, the effect of pH has been carried out by varying the solution pH from 2–11 using 0.1 N HCl and NaOH at a constant dose and dye concentration. The effect of pH on the removal of RB19 dye has been shown in Fig. S4.† The results suggest that the removal efficiencies increase with a decrease in pH values and showing highest removal at pH 2.^55,56^ This increase in removal efficiency is attributed mainly due to electrostatic attraction between negatively charged sulfonate group (SO_3_^−^) of the RB19 and positively charged ZrAC and AC. RB19 is anionic dye species, so exists in the form of negatively charged ions when dissociated in water.^59^ So, the increase in pH leads to competition between the dye anions and hydroxyl ions for the adsorption sites. Other studies observed similar results.^55,56^

#### 3.3.2. Adsorption isotherms

**Fig. 8:**
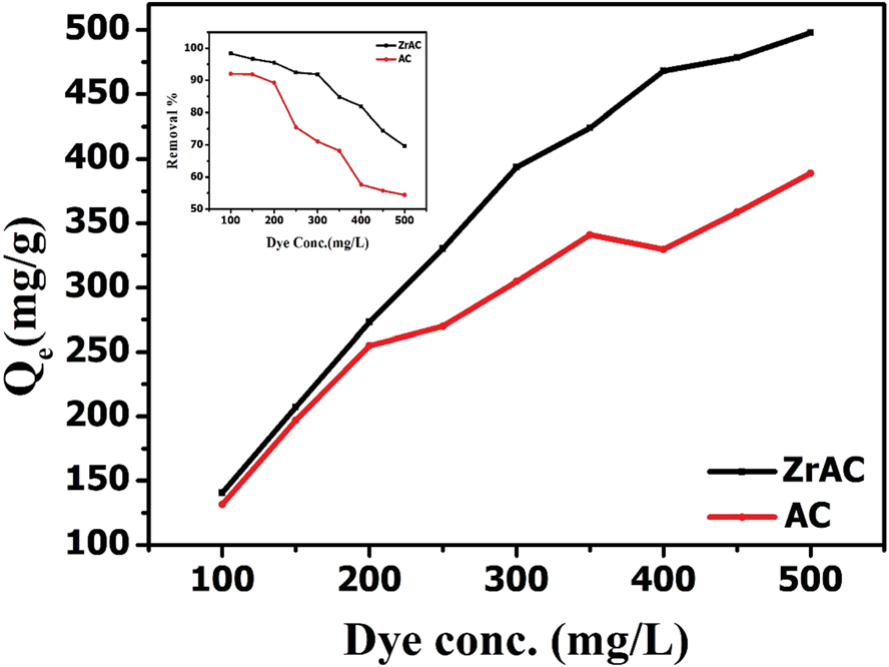
Showing maximum adsorption capacity of both the adsorbent (inset showing removal effect of both the material over reactive blue 19 dye removal).

A preliminary batch experiment has been conducted to investigate the performance of newly prepared ZrAC and to establish its viability in the wastewater treatment. The adsorption of the dye using AC and ZrAC are presented in Fig. 8. The graph exhibited that ZrAC has higher removal efficiency for reactive blue 19 dye as the concentration increases from 100 mg L^−1^ to 500 mg L^−1^. Similarly, the adsorption capacity also showed the maximum adsorption capacity for ZrAC than AC. This rise in removal efficiency and adsorption capacity of ZrAC is mainly due to the higher oxidative functionality that readily uptake the dye molecules from the solution.

The different isothermal behaviour of the adsorbents is shown in Table 3 and the experimental data were reasonably fitted with Sips and Langmuir model for ZrAC and AC, respectively. The ZrAC shows more closeness towards the Sips model based on minimal deviation from the integral equation, as suggested by higher *R*^2^. This indicates that the surface of the ZrAC seems to be energetically heterogeneous while adsorbing the dye molecules and the adsorption process follows the Freundlich isotherms during lower concentration range, followed by Langmuir isotherm at higher concentration range. The value of heterogeneity factor, *n* of sips isotherms is found to be greater than 1, suggesting some degree of heterogeneity of ZrAC-dye molecules. The monolayer adsorption capacity of ZrAC and AC were found to be 506.23 mg g^−1^ and 375.25 mg g^−1^ respectively. The maximum monolayer adsorption capacity of the ZrAC was compared with another developed adsorbent and presented in Table 4. From the table, it can easily be inferred that the ZrAC exhibits higher adsorption capacity as compared to other reported sorption capacity for the Reactive blue 19 dye.^57,58^ This could be due to its developed meso–micropores structure on the surface of ZrAC and possible enhancement of oxidative functionalities and positive charge gained because of Zr(iv) that imparts the electropositive character to the activated carbon resulting into more uptake of anionic dye molecules. The *R*_L_ values for both the adsorbent were found in between 0 to 1, thus indicating the favourable adsorption process. Hence it has been lastly corroborated that the textural and surface modification over ZrAC has been extended its applicability for the adsorption of reactive dyes (Fig. S5†).

**Table 3:** Equilibrium isotherm parameters in the adsorption process of ZrAC and AC

Model	Parameters	AC	ZrAC
Langmuir	*Q*_m_ (mg g^−1^)	375.25	506.23
	*R* ^2^	0.976	0.974
	*K*_L_ (L mg^−1^)	0.06	0.14
	*R*_L_	0.03–0.13	0.01–0.06
Freundlich	*K*_F_ (mg g^−1^)	104.86	160.02
	N	0.24	0.24
	*R* ^2^	0.92	0.94
Sips	*q*_max_ (mg g^−1^)	399.07	582.80
	*K*_S_ (mg L^−1^)^−1/*n*^	0.06	0.094
	1/*n*	0.81	0.67
	*R* ^2^	0.96	0.99

**Table 4:** Comparative study of adsorption equilibrium conducted on reactive blue dye

Dye	Adsorbent	Adsorbent dose	Removal%	Maximum adsorption capacity (mg g^−1^)	Detention time	References
Remazol brilliant blue R	Pomegranate peel activated carbon	0.1 g in 100 mL	—	370.86 mg g^−1^	—	59
Reactive blue 4	Pine sawdust (Ps) and pine sawdust–calcium chloride Ps–C–Ca solution (1 M) as impregnant	0.1 g	—	4.6 mg g^−1^ onto C–Ps and 48.6 mg g^−1^ onto C–Ps–Ca for dye conc. 1500 mg L^−1^	—	60
Remazol brilliant blue R	Mesoporous activated carbon prepared from sludge of industrial laundry	0.020 g	—	33.47 mg g^−1^ for 5 to 100 mg L^−1^ dye conc.	4 h	58
Reactive blue 19	Magnetic nanocomposite of chitosan/SiO_2_/CNTs	—	—	97.08 mg g^−1^	—	61
RBBR	Poly[2-hydroxy-3-(1-naphthyloxy)propyl]methacrylate polymer	0.04 g/50 mL	—	238.10 mg g^−1^ for dye conc. 60.85 mg L^−1^	59.91 min	62
Reactive blue 19	Bisaldehyde-functionalized silica gel	0.4 g L^−1^	—	72.99 mg g^−1^ for dye conc. 20 mg L^−1^	30 min	55
Reactive blue 19	Lignocellulosic waste	0.25 g/50 mL	—	71.6 mg g^−1^ for dye conc. 500 mg L^−1^	240 min	56
Reactive blue 19	ZrAC	0.07 g L^−1^	96% for 250 mg L^−1^ dye conc.	506.23 mg g^−1^ for dye concentration of 500 mg L^−1^	90 min	This study

### 3.4. Kinetic and thermodynamic studies

To evaluate the dye adsorption rate of ZrAC and AC, the adsorption kinetics were performed, and the results are shown in Table 5. Out of three kinetic models, the *R*^2^ value of the pseudo-second-order kinetic model was found more closer to unity suggesting this model fitted well for describing the rate of adsorption in ZrAC and AC for temperature range. A slight increase in sorption capacity, with an increase in temperature, was observed for both ZrAC and AC. The value of *h* of ZrAC was found higher than AC indicating the faster rate of removal of dye by ZrAC. The intraparticle diffusion was used to describe the diffusion mechanism of RB19 into the adsorbents. The adsorption process follows various steps and out of which the slowest step affects the rate of the adsorption process. The graph (Fig. S6†) of intraparticle diffusion model indicates that the straight lines did not pass through the origin, implying that the film diffusion and intraparticle diffusion are involved in the adsorption process, but it is not the rate-controlling step. Also, it was observed that *C* ≠ 0, revealing that the reaction process is a quite complex process and it is not entirely controlled by an intraparticle diffusion model.

**Table 5:** Kinetic parameters in the adsorption process of AC and ZrAC

Adsorbent	Parameters	Temperature (K)		
		298	308	318
AC	*q*_e_ (exp) (mg g^−1^)	133.761	135.384	138.007

	**Pseudo first order**			
	*q*_e,1_ (cal) (mg g^−1^)	163.008	151.775	163.842
	*t* ^1/2^	15.97	15.57	13.53
	*R* ^2^	0.930	0.911	0.964
	*k*_1_ × 10^3^ (min^−1^)	43.4	44.5	51.2
	SSE	13 099.6	6058.29	10 665.8

	**Pseudo second order**			
	*q*_e,2_ (cal) (mg g^−1^)	166.667	158.73	163.934
	*t* ^1/2^	24.48	18.30	17.19
	*H*	6.807	8.673	9.532
	*R* ^2^	0.986	0.991	0.991
	*k*_2_ × 10^3^ (g mg^−1^ min^−1^)	0.25	0.34	0.35
	SSE	268.78	243.34	444.88

	**Intraparticle diffusion model**			
	*R* ^2^	0.979	0.978	0.945
	*k*_i_ (mg g^−1^ min^−0.5^)	11.536	10.191	10.281
	*C*	24.107	38.371	42.721
ZrAC	*q*_e_ (exp) (mg g^−1^)	141.654	142.43	144.79

	**Pseudo first order**			
	*q*_e,1_ (cal) (mg g^−1^)	143.30	146.70	155.57
	*t* ^1/2^	15.7	14.11	13.78
	*R* ^2^	0.934	0.919	0.910
	*k*_1_ × 10^3^ (min^−1^)	43.9	49.1	50.3
	SSE	1770	2379.04	4794.17

	**Pseudo second order**			
	*q*_e,2_ (cal) (mg g^−1^)	163.93	161.29	163.93
	*t* ^1/2^	16.032	13.26	13.29
	*H*	10.22	12.16	12.33
	*R* ^2^	0.995	0.997	0.993
	*k*_2_ × 10^3^ (g mg^−1^ min^−1^)	0.38	0.46	0.45
	SSE	197.68	220.17	440.86

	**Intraparticle diffusion model**			
	*R* ^2^	0.974	0.967	0.977
	*k*_i_ (mg g^−1^ min^−0.5^)	10.062	9.298	9.017
	*C*	46.424	55.689	59.664

The thermodynamics properties were also investigated to determine the core thermodynamic behaviour of the adsorbents by calculating parameters such as Δ*G*°, Δ*H*° and Δ*S*° (Table 6). The negative values of Δ*G*° for both the adsorbents, indicates that all the adsorption process is spontaneous and thermodynamically favourable. It has been observed that the gradual decrease in Δ*G*° with an increase in temperature indicates that the adsorption is favoured at a higher temperature that is well supported for dye wastewater treatment as the effluent discharge from dye industry contain higher temperature. The positive values of Δ*H*° for both cases also suggested that the reaction is endothermic in nature. Also, as the heat input is higher, the forward reaction is favourable as the products of the reaction consume heat. Since the Δ*H*° value was found in between 20 to 80 kJ mol^−1^ for ZrAC, it clearly demonstrated that the adsorption of dye molecules is endothermic in nature and governed *via* physisorption and chemisorption as well, that suggests the impregnation of Zr over AC boost the sorption behaviour. The values of Δ*G*° of AC governs that the physisorption was dominating the adsorption mechanism. The positive value of Δ*S*° further advocated increased randomness at the solid/liquid interface during dye adsorption. Similar, thermodynamic results were also reported by other authors.^62,63^

**Table 6:** Thermodynamics parameters of AC and ZrAC on dye adsorption

Adsorbent	Temperature (K)	*K*_c_	Δ*G*° (kJ mol^−1^)	Δ*H*° (kJ mol^−1^)	Δ*S*° (kJ mol^−1^)	*R* ^2^
AC	298	3.82154	−3.32157	15.35	0.062	0.96
	308	4.295718	−3.73254			
	318	5.165874	−4.3414			
	328	6.770551	−5.2156			
ZrAC	298	13.81591	−6.50566	29.27	0.12	0.95
	308	22.24284	−7.94338			
	318	25.84722	−8.59834			
	328	43.72352	−10.3023			

### 3.5. Desorption and regeneration

**Fig. 9:**
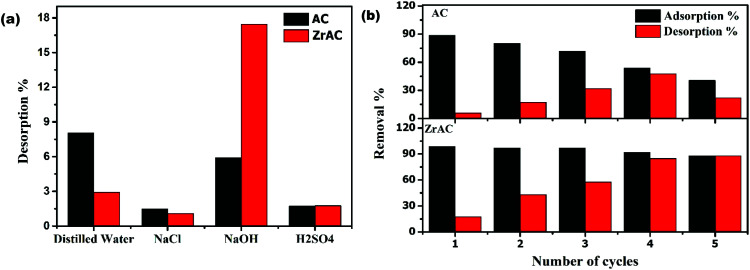
Regeneration of spent adsorbent: (a) showing desorption% of AC and ZrAC for different eluents and (b) showing adsorption% and desorption% of AC and ZrAC using NaOH as eluent.

The desorption study has been conducted to regenerate the exhausted adsorbents and restore their character. The regeneration process plays a crucial role in large scale practicing of the feasible treatment process and minimizing the amount of waste (secondary pollutant). In this work, four desorbing agents were used to regenerate the spent adsorbent. The desorption efficiency of four eluents has been investigated and presented in Fig. 9(a). It has been observed that the out of four, the regeneration capacity of ZrAC was more prominent in case of NaOH, that can easily remove the dye molecules from the surface of the adsorbent. Also, by using basic eluent as a desorbing agent, the surface of the adsorbent gets more OH^−^ ions from the solution and hence anionic exchange will occur more spontaneously, as a result of which the dye molecules get released from the surface of the adsorbents. Additionally, the change in pH values due to NaOH corroborate the easy release of RB19 from the adsorbent surface.

To regenerate the ZrAC and AC adsorbent, desorption study has been conducted for five cycles using NaOH as a desorbing agent and the results are presented in Fig. 9(b). The results clearly suggested that AC has a lower desorption rate for reactive blue 19 release from saturated AC as compared to ZrAC surface. Simultaneously, the adsorption rate as per cycle repeat goes on decreasing drastically for the case of AC while only small decrease was observed until the fifth cycle of re-adsorption for the ZrAC. Besides this, the stability of the zirconium in ZrAC has also been examined using XPS and ICP-MS data. In ICP-MS analysis of zirconium, before and after adsorption cycles, shows a positive agreement with the results showing less deviation (Table S2†). Initially, the zirconium concentration in ZrAC was 92.43 mg g^−1^ that changes to 86.49 mg g^−1^ after adsorption. This changes mainly attributed due to attachment of dye molecules over the adsorbent. Also, the XPS peak of ZrAC shows similar peaks of zirconium after the adsorption process [Fig. 7(d)]. Thus, corroborated that the regeneration capabilities of ZrAC were more prominent that finally affirms its potentially more use in a continuous flow system as reusable adsorbent.

## 4. Conclusion

In the present study, a novel material has been synthesized by using metal salt (zirconium oxychloride) as an impregnating metal on activated carbon. The developed material successfully gained the meso–microporous structures along with oxidative functionalization, because of Zr(iv) impregnation on the surface. The physicochemical and structural results suggested that the modification of activated carbon leads to widening of the pores thereby results in the transformation of micropores into meso/macropores, due to the pore blocking and metal residing in and around the pores. The textural results further validated the structural transformation into eroded and heterogeneous surface upon modification. The modification process also enhanced the oxidative functionalities as well as Zr(iv) impregnation on AC, as observed in FTIR, XRD and XPS peaks. These modifications were validated with the experimental results, and it was observed that the maximum monolayer adsorption of ZrAC was found to superior than other developed adsorbent materials. The isothermal, kinetic and thermodynamics results corroborate that the adsorption process is favourable, endothermic and spontaneous in nature. The desorption and regeneration studies finally augmented the enhanced adsorption affinity of ZrAC towards RB19. Also, the stability of zirconium over activated carbon has been examined with ICP-MS and XPS, and it was found that the ZrAC has similar stability after adsorption cycles; thus, acclaimed its reusability. Henceforth, culminating the characterization and experimental results, it has been advocated that the ZrAC has the profuse capacity to be used as a future adsorbent for the removal of reactive blue 19 dye.

## Conflicts of interest

There are no conflicts to declare.

## Supplementary Material

RA-010-C9RA10103A-s001
