# Supplementary material for: Synthesis, characterization and sorption studies of a zirconium(iv) impregnated highly functionalized mesoporous activated carbons
Source: RSC Adv. 2020 Apr 6;10(23):13783–98. doi: 10.1039/c9ra10103a (PMC9051575; doi:10.1039/c9ra10103a)
Supplement: RA-010-C9RA10103A-s001 [file RA-010-C9RA10103A-s001.pdf]

Electronic Supplementary Material (ESI) for RSC Advances.
This journal is © The Royal Society of Chemistry 2020

Supplementary Data

Synthesis, characterization and sorption studies of Zirconium (IV) impregnated highly functionalized mesoporous sorbent

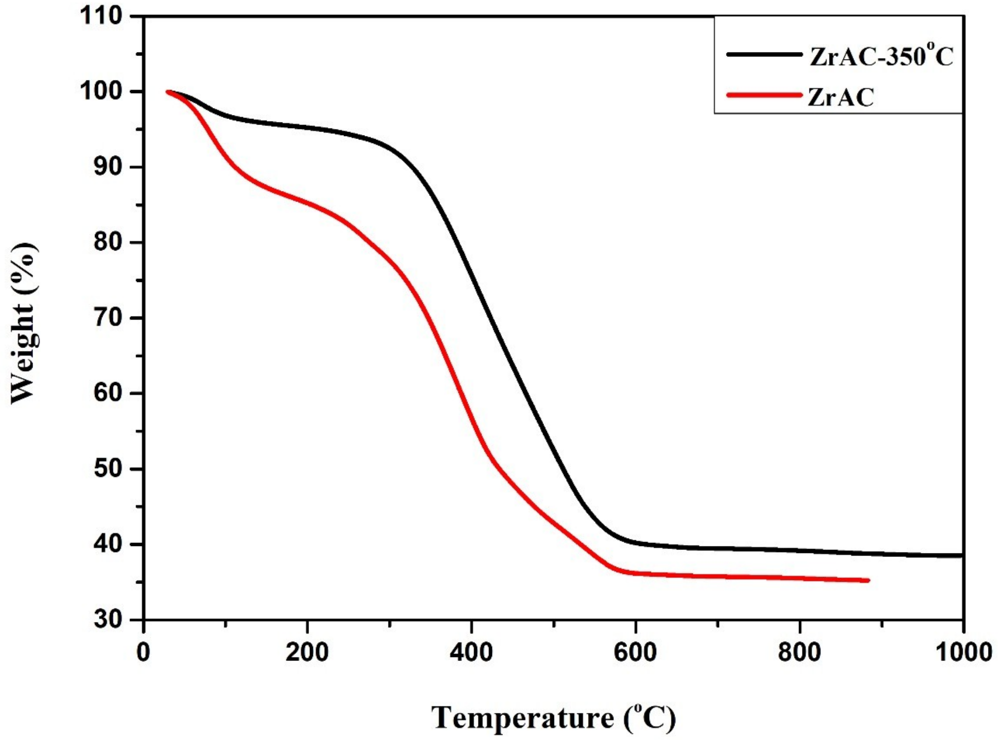

Figure S1: Thermal characteristics of heat-treated ZrAC_350^{0}C and ZrAC.

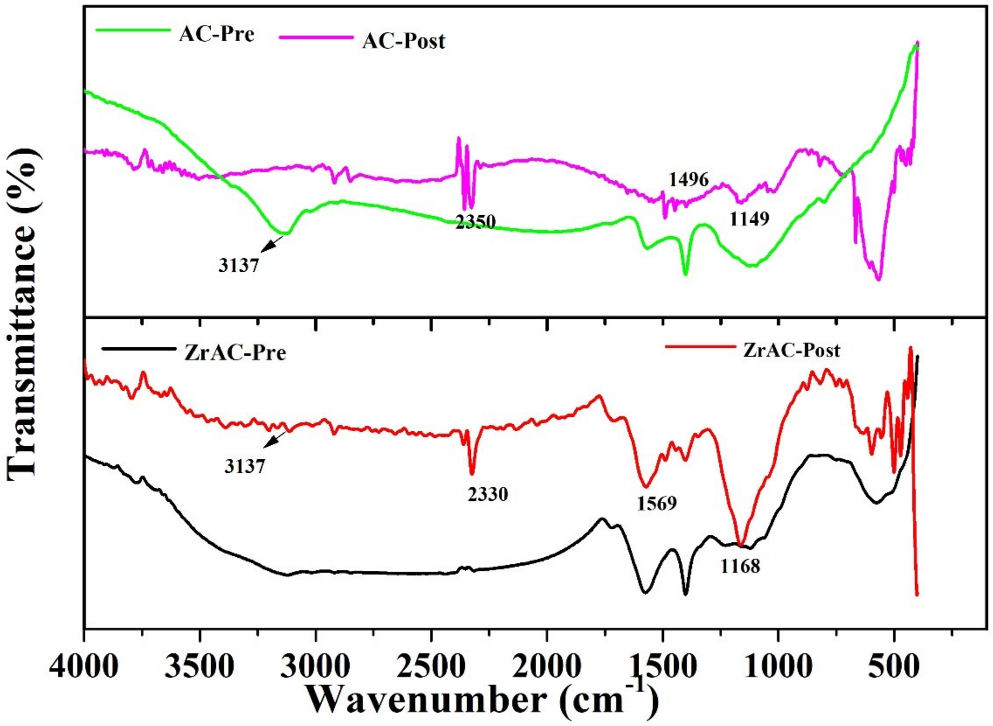

Figure S2: FTIR spectra of AC and ZrAC after adsorption of Reactive Blue 19

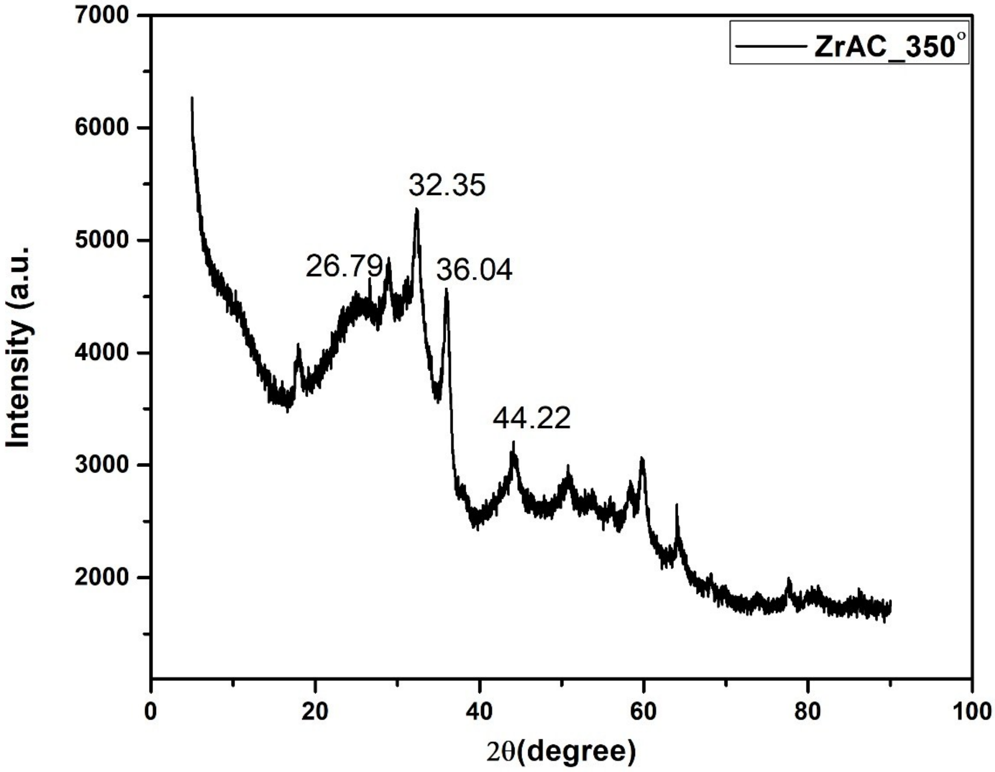

Figure S3: XRD diffraction pattern for heat-treated ZrAC at 350^{0}C (ZrAC_350^{0}C) and ZrAC.

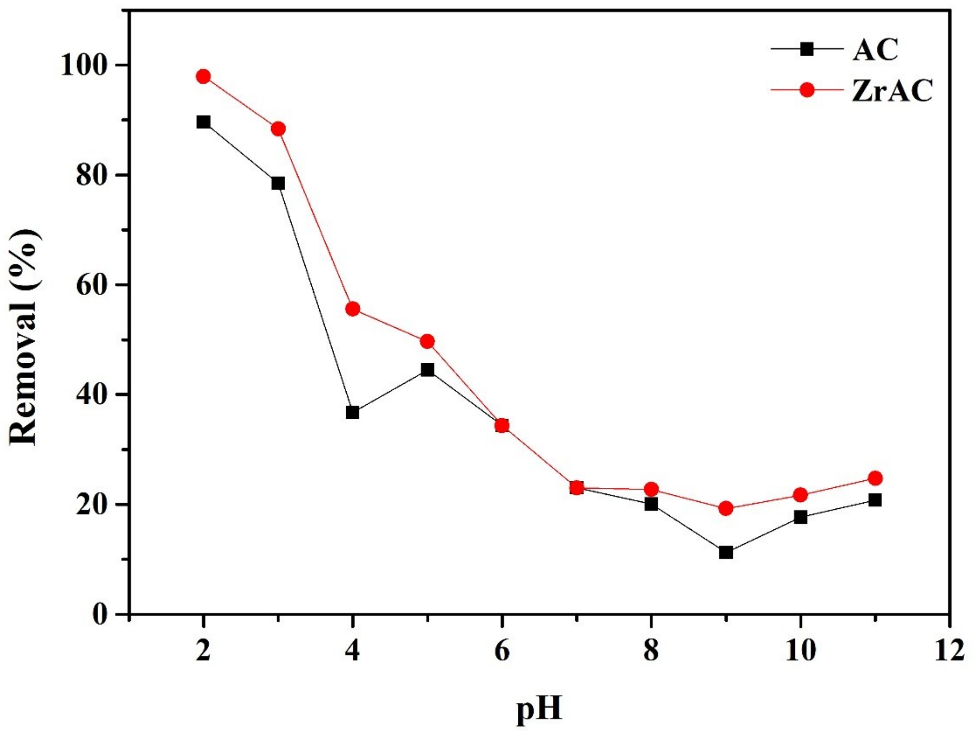

Figure S4: Effect of pH on the removal of Reactive Blue 19

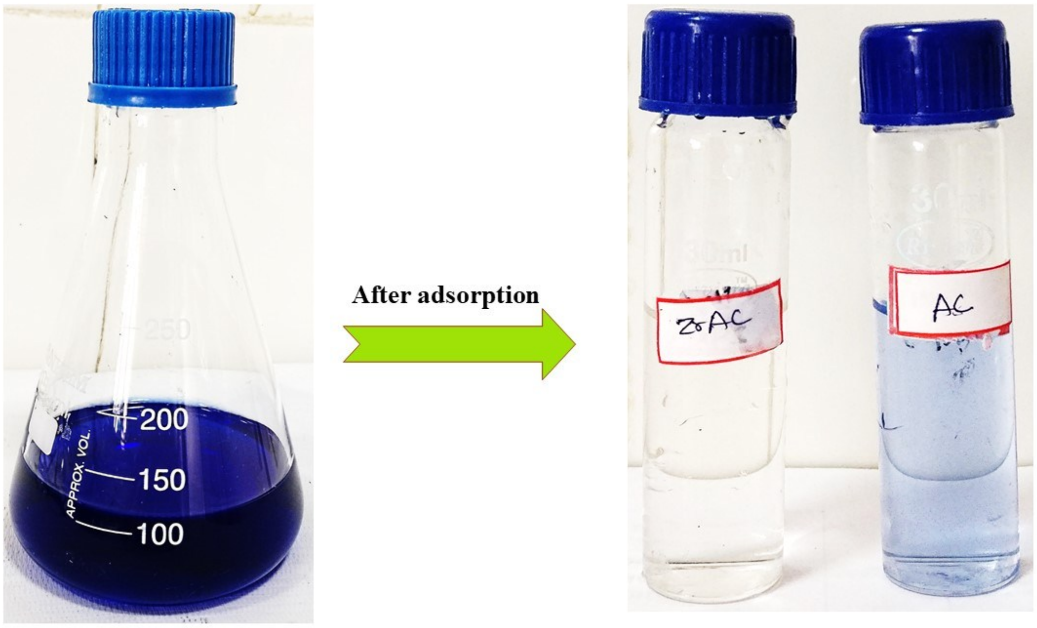

Figure S5: Experimental result: Adsorption difference of AC and ZrAC

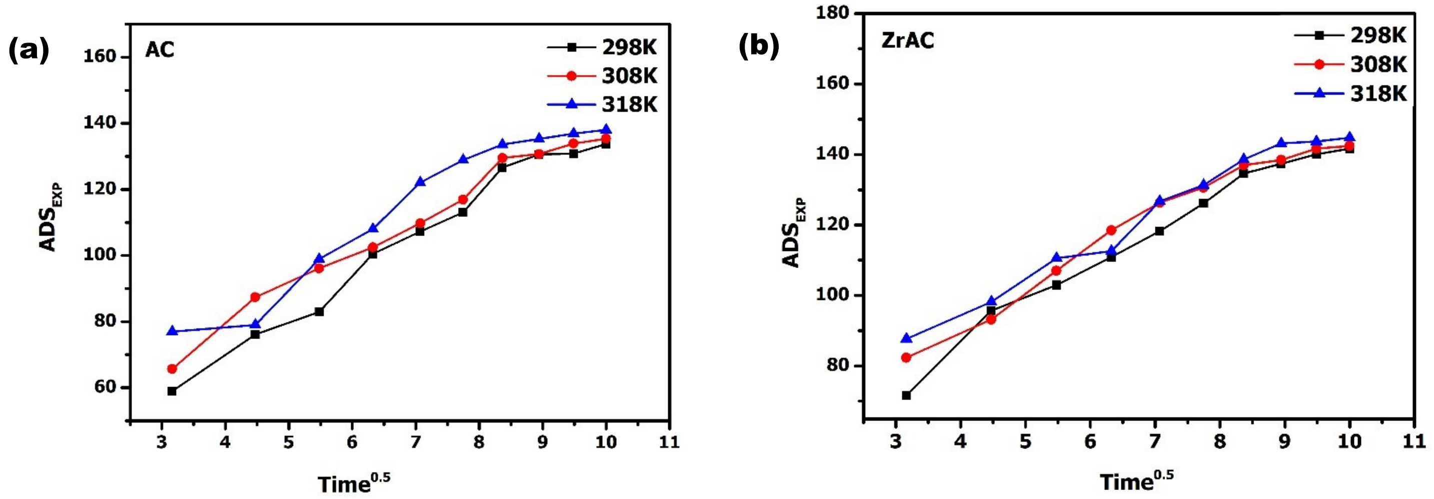

Figure S6: Intraparticle diffusion model of (a) AC and (b) ZrAC.

Table S1: EDX composition (weight %) of the elements present in AC and ZrAC.

Samples	Weight (%) of elements						
	**C**	**O**	**Cl**	**K**	**Mn**	**Zr**	**Others including Si, N, etc**
**AC**	85.33	12.56	-	0.68	-	-	1.53
**ZrAC**	49.85	25.25	0.12	0.17	5.43	14.11	5.07

Table S2: Concentration of Zirconium on ZrAC before and after the adsorption using ICP-MS

Adsorbent	Zr Concentration (mg/gm)
ZrAC-Pre	92.43 ±5
ZrAC-Post	86.49±5
